# Supplementary material for: Peak learning of mass spectrometry imaging data using artificial neural networks
Source: Nat Commun. 2021 Sep 20;12:5544. doi: 10.1038/s41467-021-25744-8 (PMC8452737; doi:10.1038/s41467-021-25744-8)
Supplement: Supplementary file 1 — Supplementary Information [file 41467_2021_25744_MOESM1_ESM.pdf]

Supplementary Information

Peak Learning of Mass Spectrometry Imaging Data Using Artificial Neural Networks

Walid M. Abdelmoula[1], Begona Gimenez-Cassina Lopez[1], Elizabeth C. Randall[2], Tina Kapur[2], Jann N. Sarkaria[3], Forest M. White[4], Jeffrey N. Agar[5], William M. Wells[2,6], and Nathalie Y.R. Agar*[,1,2,7]

[1]Surgical Molecular Imaging Laboratory, Department of Neurosurgery, Brigham and Women's Hospital, Harvard Medical School, Boston, MA 02115, USA
[2]Department of Radiology, Brigham and Women's Hospital, Harvard Medical School, Boston, MA 02115, USA
[3]Department of Radiation Oncology, Mayo Clinic, 200 First St SW, Rochester MN 55902, USA
[4]Department of Biological Engineering, Koch Institute for Integrative Cancer Research, MIT, Cambridge, MA, 02142, USA
[5]Department of Chemistry and Chemical Biology, Northeastern University, 412 TF (140 The Fenway), Boston, MA 02111, USA
[6]Computer Science and Artificial Intelligence Laboratory, MIT, Cambridge, MA 02139, USA
[7]Department of Cancer Biology, Dana-Farber Cancer Institute, Harvard Medical School, Boston, MA 02115

This file includes:

- **Supplementary Methods**
- **Supplementary Figures 1 to 13**
- **Supplementary Tables 1 to 8**

Supplementary Methods

MSI datasets from five different tissue types were analyzed using the neural network model and their description is as follows.

1- 2D MALDI FT-ICR MSI of a human prostate dataset

The analysis was performed according to Randall *et al*[1]. Briefly, 12 μm thickness prostate sample diagnosed with a Gleason score of (3+4)=7 were mounted on a microscopy glass slide and coated with CHCA (5 mg/mL in 70/30 methanol/water with 0.1% trifluoroacetic acid V/V) using an automated sprayer (TM-Sprayer, HTX Imaging, Carrboro, NC). The analysis of the samples was performed on a 9.4 Tesla SolariX XR FT ICR mass spectrometer (Bruker Daltonics, Billerica, MA) using the MALDI source in positive ion mode in the mass range between 250-1000 *m/z*, with a spatial resolution of 120 μm. Internal online calibration was performed using heme *m/z* 616.1776 during data acquisition.

2- 3D MALDI FT-ICR MSI dataset of a PDX mouse brain model of glioblastoma

The intracranial tumor belonging to a PDX model of GBM12 (PDX National Resource, Mayo Clinic), was analyzed by MALDI FT ICR MSI using a 9.4 Tesla SolariX mass spectrometer (Bruker Daltonics, Billerica, MA), using continuous accumulation of selected ions in the mass range between 380-620 *m/z*. The indium tin oxide (ITO)-coated slide with 12 μm thickness tissue sections , was coated with DHB (160 mg/mL in a 70/30 v/v solution of methanol/0.2% TFA), according to Randall *et al*[2]*.* The 3D MSI dataset was collected from 4 tissue sections with an inter-slice distance of 160 μm. Internal online calibration was performed using heme *m/z* 616.1776 during data acquisition.

3- 3D DESI MSI dataset of human colorectal adenocarcinoma

A clinical volumetric tissue specimen of colorectal adenocarcinoma was sliced into 26 sections each of 10 μm thickness and imaged by DESI MSI (Thermo Exactive instrument, Thermo

Scientific GmbH, Germany). This 3D MALDI MSI dataset was acquired in the negative-ion mode and covered a mass range of *m/z* 200-1050. The acquisition spatial resolution was set to 100 µm, and the full 3D MSI dataset encompasses 148044 spectra each of 8073 dimensions. This 3D MSI dataset is publicly available and for more information on sample preparations and acquisition details we refer to Oetjen *et al*[3].

4- 3D MALDI MSI dataset of human oral squamous cell carcinoma

A volumetric tissue specimen of human oral squamous cell carcinoma was sliced into 58 consecutive sections each of 10 µm thickness and imaged by MALDI MSI (Autoflex speed™, Bruker Daltonics, Germany). This 3D MALDI MSI dataset was acquired in the positive-ion mode and covered a mass range of *m/z* 2,000-20,000. The acquisition spatial resolution was set to 60 µm, and the full 3D MSI dataset encompasses 825558 spectra each of 7665 dimensions. This 3D MSI dataset is publicly available and for more information on sample preparations and acquisition details we refer to Oetjen *et al*[3].

5- 3D MALDI MSI data of mouse kidney

A volumetric tissue specimen of mouse kidney was sliced into 73 consecutive sections each of 3.5 µm thickness and imaged by MALDI MSI (Autoflex speed™, Bruker Daltonics, Germany). This 3D MALDI MSI dataset was acquired in the positive-ion mode and covered a mass range of *m/z* 2,000-20,000. The acquisition spatial resolution was set to 50 µm, and the full 3D MSI dataset encompasses 1,362,830 spectra each of 7671 dimensions. This 3D MSI dataset is publicly available and for more information on sample preparations and acquisition details we refer to Oetjen *et al*[3].

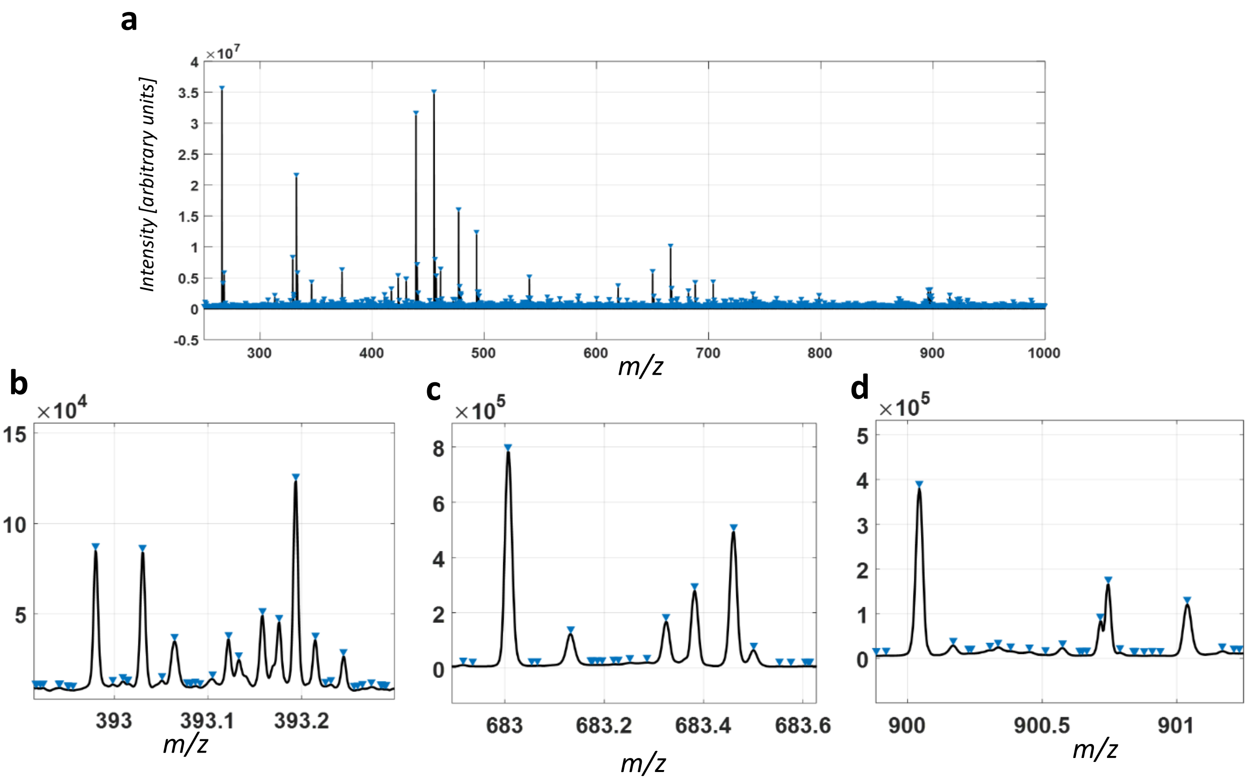

Supplementary Figure 1. FT-ICR MSI prostate cancer dataset: local maxima are highlighted in the mean spectrum(a), and a zoomed-in regions within the mean spectrum (b-d). The identified local maxima significantly reduce the original spectral dimensions from about 0.73 million to 61,343 *m/z* features. This impacts the spectral sparsity but not the spectral representation.

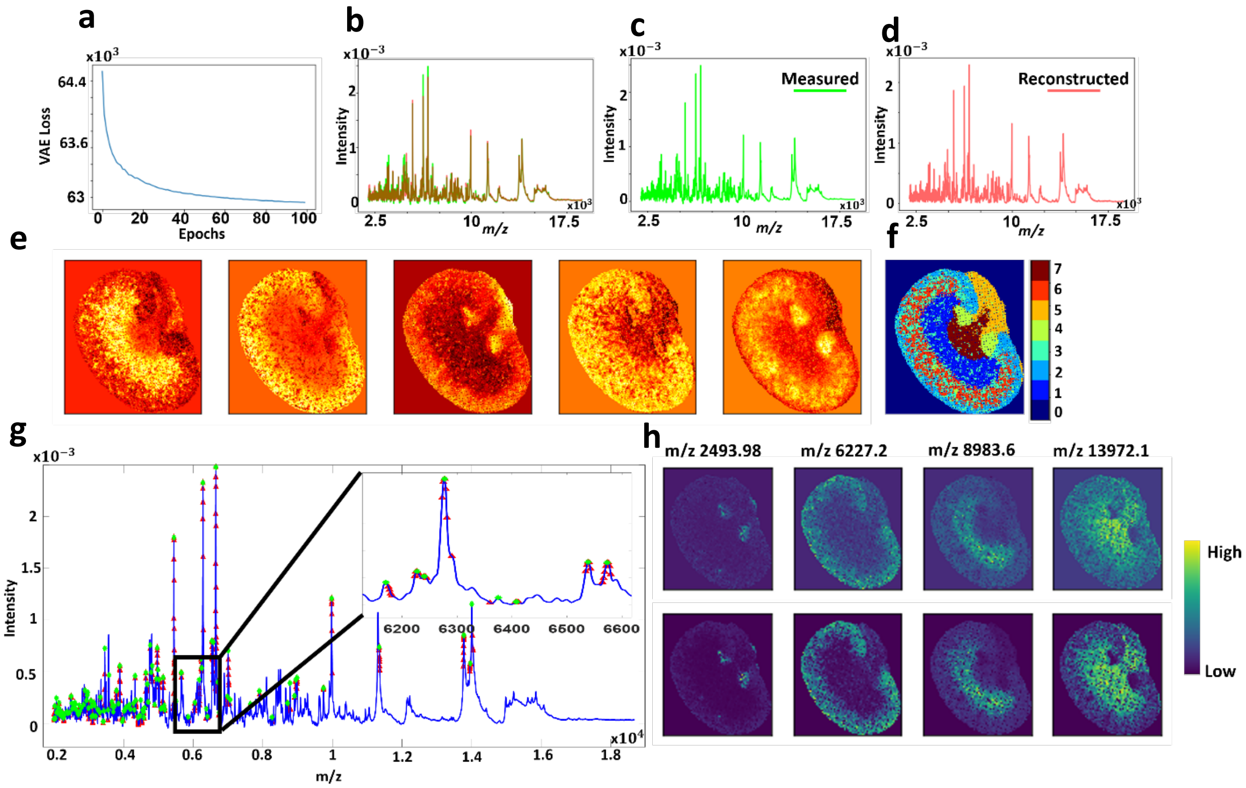

Supplementary Figure 2. Analysis of a training 2D MALDI MSI dataset of a mouse Kidney: **a.** convergence distribution, **b.** Overlay of the TIC normalized original mean spectrum and the estimated average spectrum (**c**-**d**) with an overall mean squared error of 5.5×10^{-3}. **e**. The low dimensional encoded features capture molecular patterns that were segmented using GMM (k=7) as shown in (**f**). **g.** The set of learned *m/z* variables that were strongly linked to the identified patterns are highlighted in the mean spectrum as such: *m/z* bins (red points) and their associated m/z peaks (green points). **h**. Spatial distribution of a few learned m/z features co-localized with some identified spatial patterns, and the close similarity of their distributions for original (top row) and reconstructed data (bottom row) reflects high unsupervised learning quality.

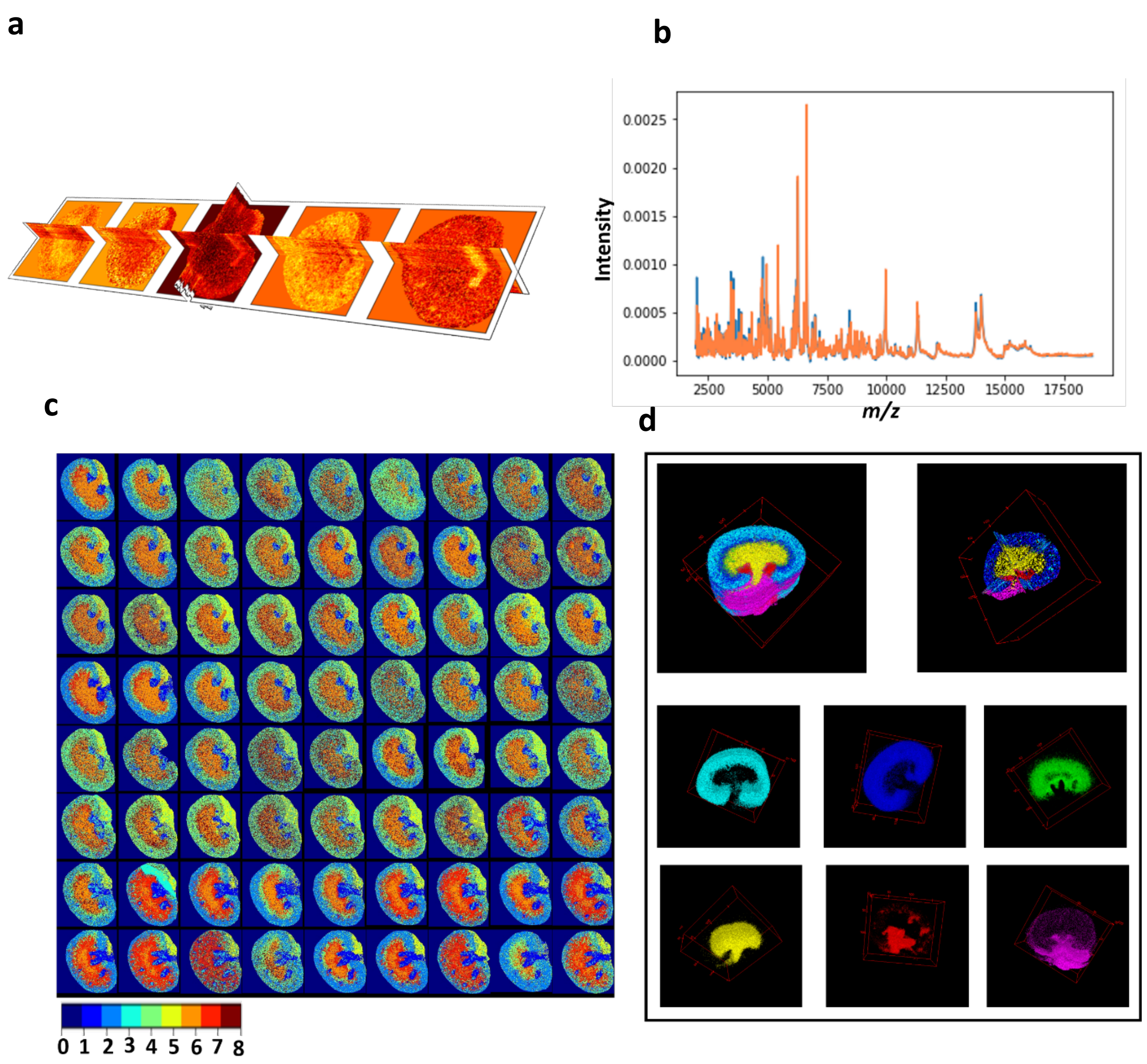

Supplementary Figure 3. 3D MALDI MSI dataset from 72 mouse kidney tissue samples that were withheld for test analysis using the trained model shown in the main manuscript Figure 5: a. 3D distribution of the encoded features, b. overlay of the overall mean spectrum of both TIC normalized original (blue) and reconstructed (orange) full test dataset with mean squared error of 3.11×10^{-3}. **c**. clustering of encoded features using GMM (k=8) reveals molecular patterns that form anatomical structures that are volumetric rendered in (d).

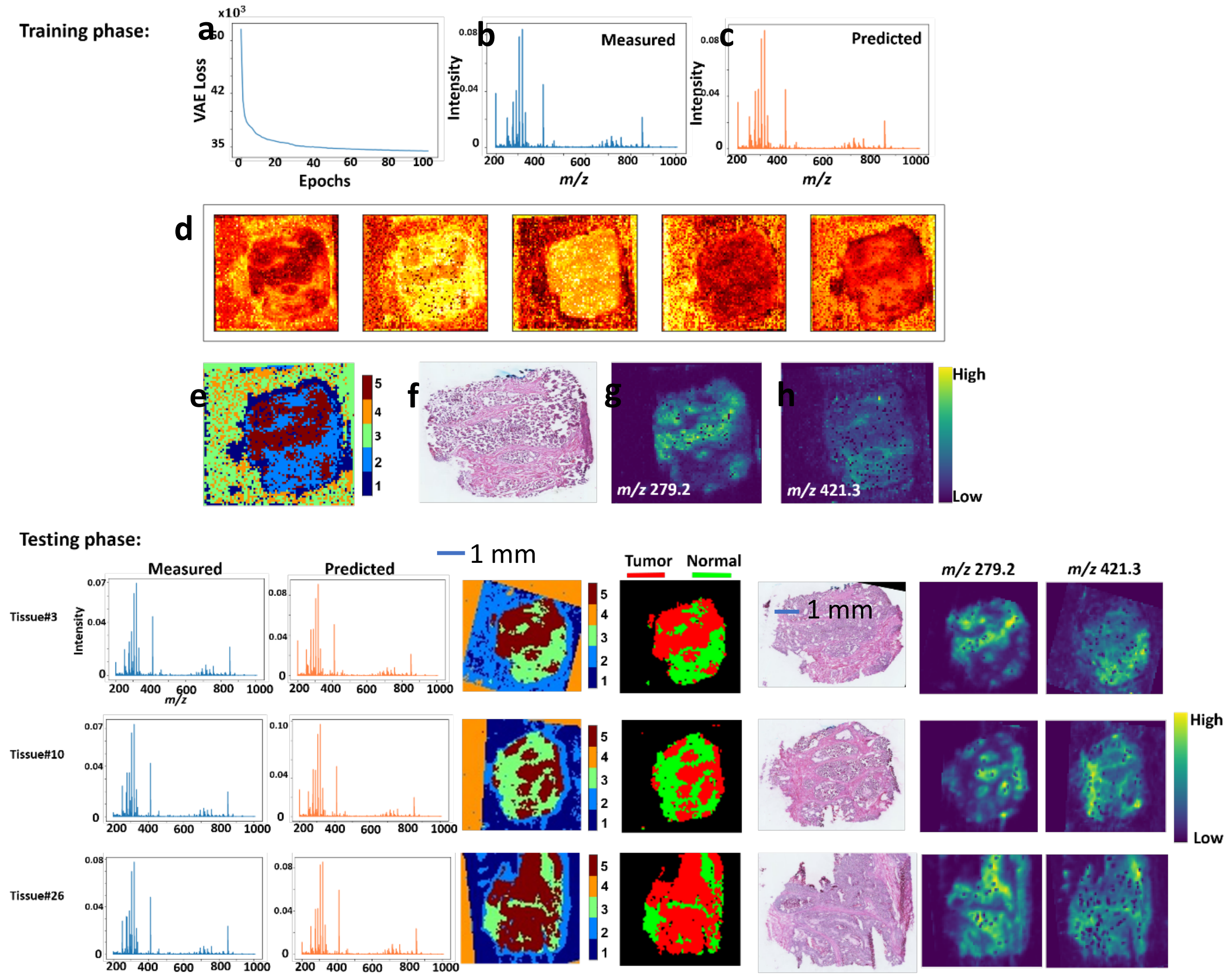

Supplementary Figure 4. Deep-Learning based analysis of a 3D DESI MSI dataset of a clinical specimen of colorectal carcinoma. In the **training phase** the VAE model took around 3.2 minutes to run on a training sample of 5694 spectra each of 8073 dimensions: **a.** distribution of the optimization convergence with the number of iterations (epochs), average TIC-normalized spectra of both measured (b) and predicted (c) spectral data are closely distributed, d. captured encoded features that visualizes a non-linear embedding of original data, e. these encoded features were clustered with Gaussian mixture model (with k=5) and it revealed two biologically-relevant tissue types that reconcile with the H&E histology (**f**), namely: tumor region (cluster#5) and connective tissue (cluster#2), and distribution of ion features at m/z 279.23 ± 0.1 (**g**) and m/z 421.31 ± 0.1 (**h**) were found elevated and highly correlated with the tumor and connective tissue clusters, respectively. In the **testing phase** the trained model was applied on the withheld test dataset: results on three distinct test MSI datasets sampled from different locations within the volumetric tissue specimen, each sample was analyzed within 4 seconds in which the model accurately predicted the measured data (overall mean squared error of 1.77×10^{-4}), and molecular-based tumor and connective tissue clusters were identified and found in close agreement with the H&E histology.

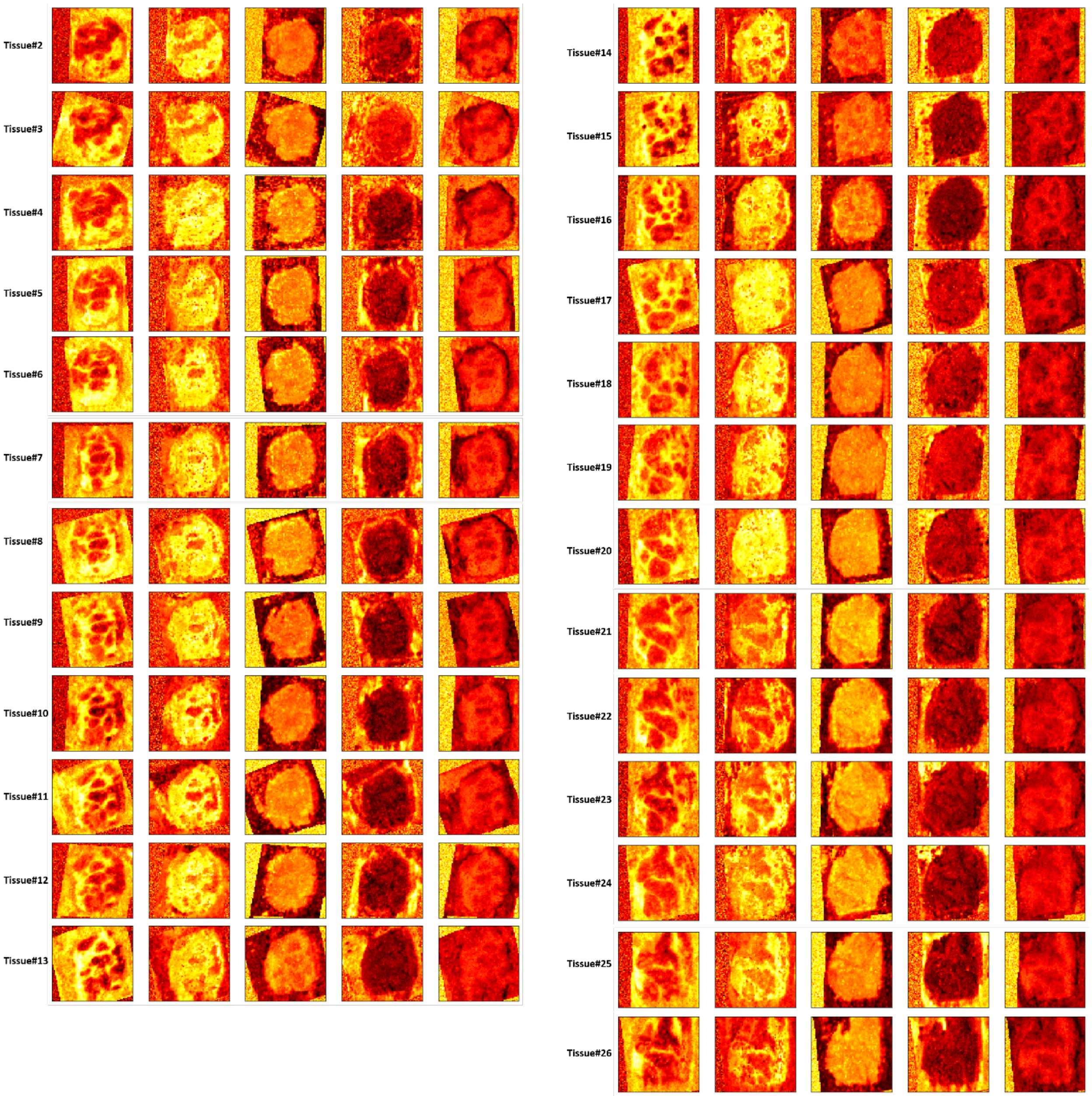

Supplementary Figure 5. Encoded features of 3D DESI MSI test dataset of 25 tissue sections of colorectal adenocarcinoma. High dimensional spectral data of each tissue section were non-linearly mapped into a smaller subspace represented by 5 encoded features. The encoded features reveal structural information that depict molecular patterns from original high dimensional data.

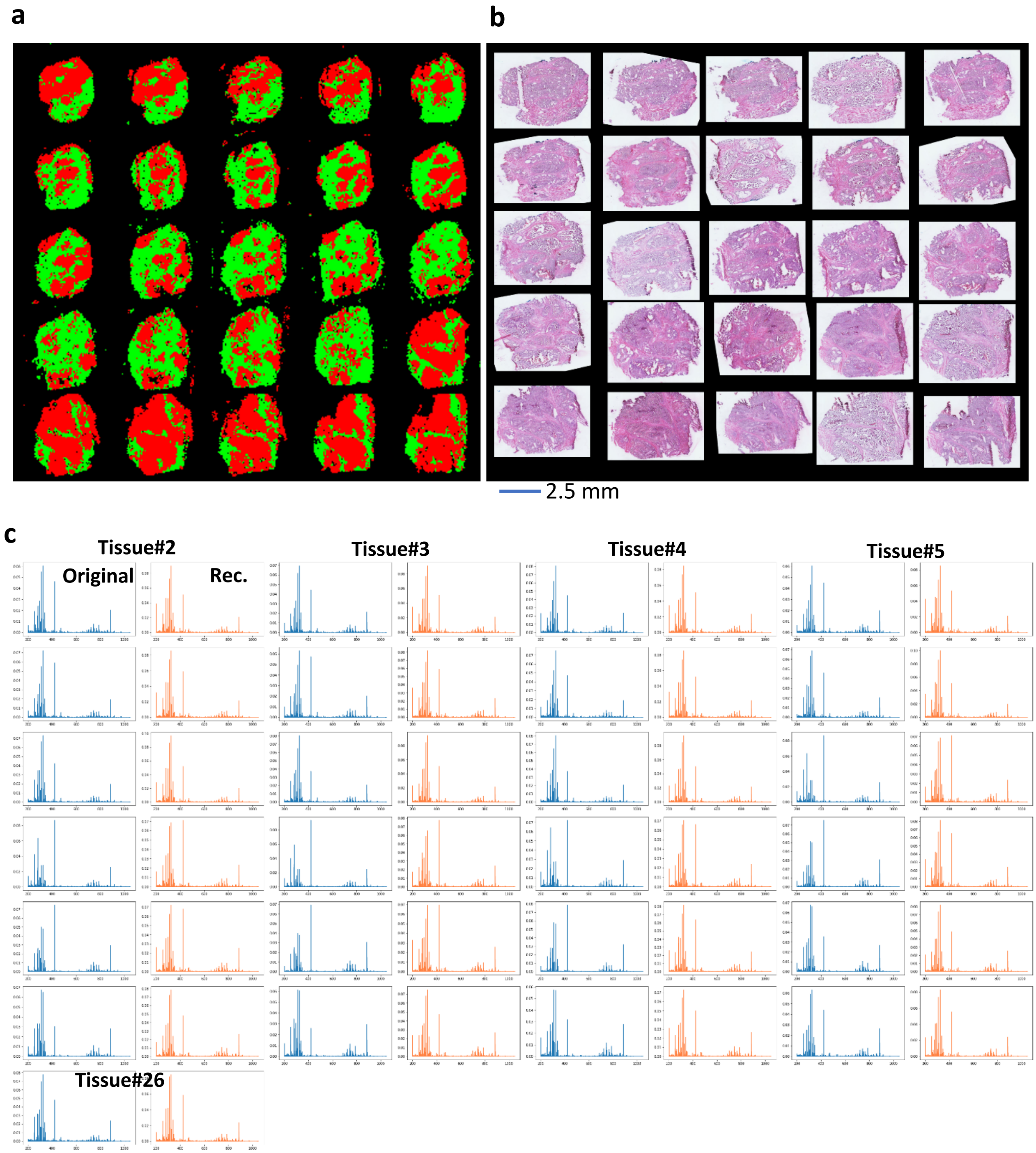

Supplementary Figure 6. Analysis of 3D DESI MSI test dataset of colorectal adenocarcinoma: **a.** Tumor (red) and connective tissue (green) clusters were extracted from the clustered image of Gaussian mixture model (k=5) on the encoded features shown in Figure S4, and they appear in a close agreement with **b.** their counterparts H&E histology. **c.** Distribution of average spectrum for both TIC normalized original (blue) and reconstructed (orange) MSI data.

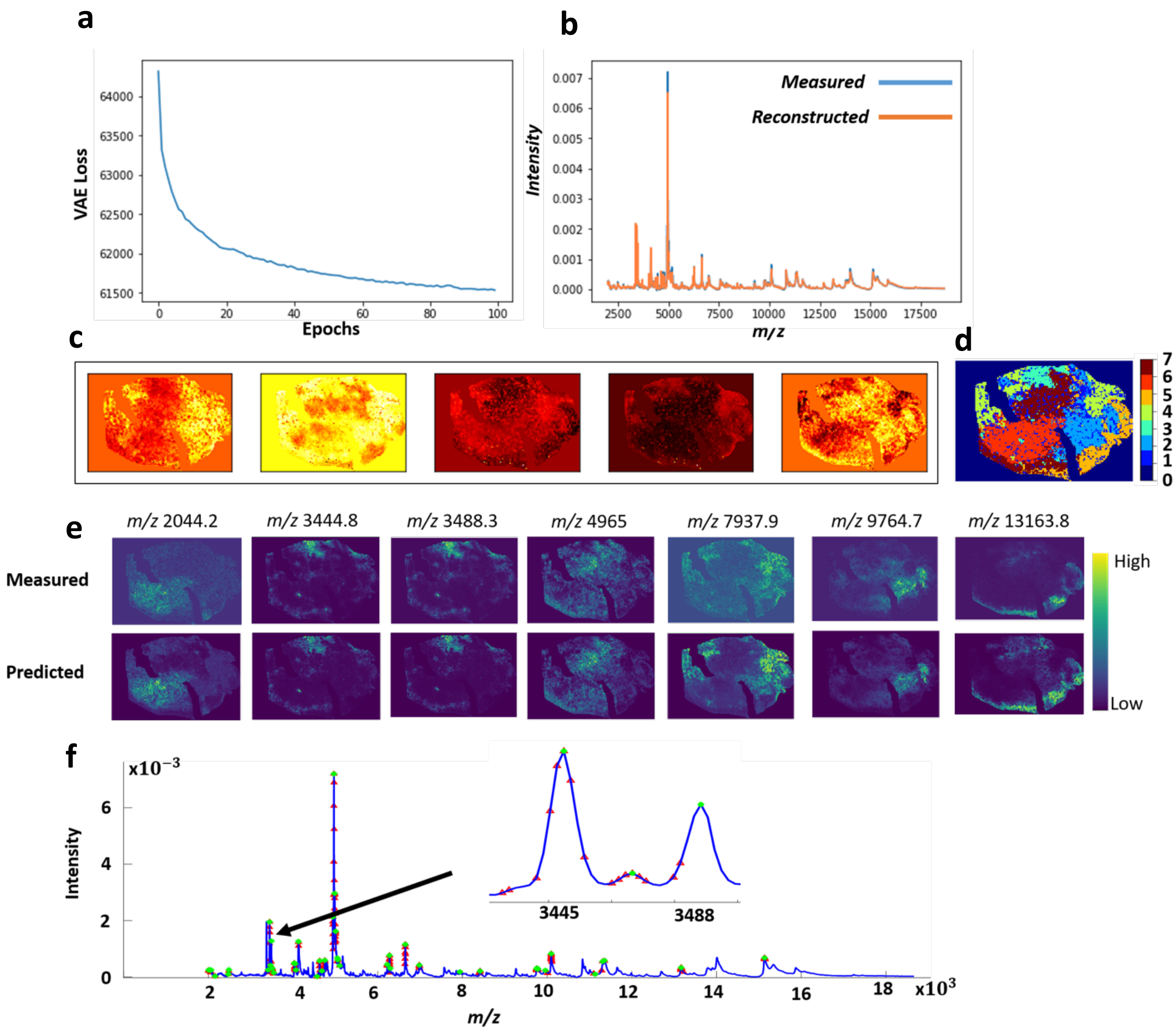

Supplementary Figure 7. Deep learning analysis of a 2D MALDI MSI dataset of OSCC tissue: **a.** Optimizer convergence, **b.** Overlay of the mean spectrum distribution of TIC normalized original (blue) and reconstructed (orange) MSI data. **c.** Encoded features of 5 dimensions capture molecular patterns from original high dimensional data and were clustered by the GMM (k=7) to reveal clusters of molecularly distinct regions (**d**). Each of these clusters was correlated with the reduced MSI data of selected peaks (green points in panel **f**), and the distribution of highly correlated m/z peaks in each cluster is given in (**d**). The spatial distributions of m/z images (**e**) confirm high quality of reconstructed data. f. High-weighted unbanned m/z variables underlying the molecular structures captured by the encoded features are highlighted in the mean spectrum: m/z bins (red points) and their parent peaks (green points).

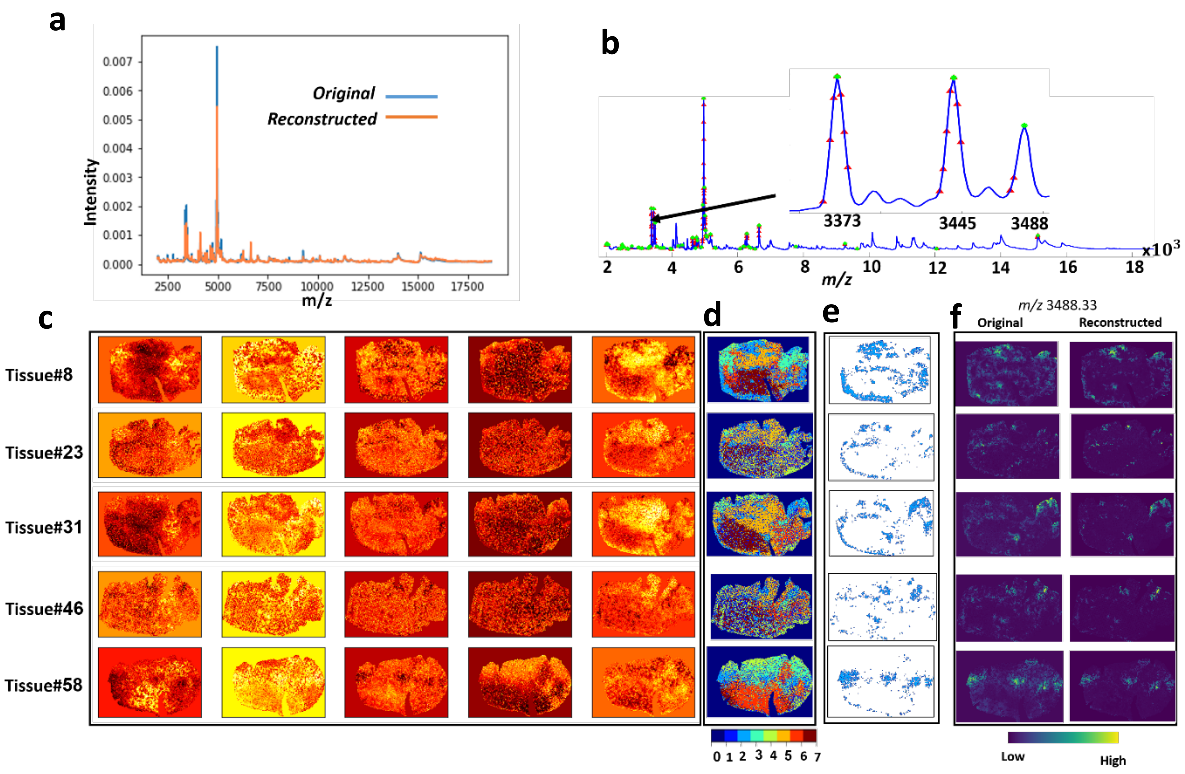

Supplementary Figure 8. Analysis of test MSI dataset from 5 tissue sections that were sampled from different locations within the volumetric tissue specimen: a. Overlay of original and estimated TIC normalized mean spectrum, and b. highlights m/z variables (red points highlight m/z bins whereas green points highlight m/z peaks) underlying the molecular patterns depicted in the latent space (c). These molecular patterns were clustered by the GMM (d) and the cluster#2 was extracted (e) and found highly correlated with the defensins produced by the Neutrophils, namely lipid ions at m/z 3373, 3445, and 3488. One of these ions was spatially mapped (f) for both original and reconstructed data.

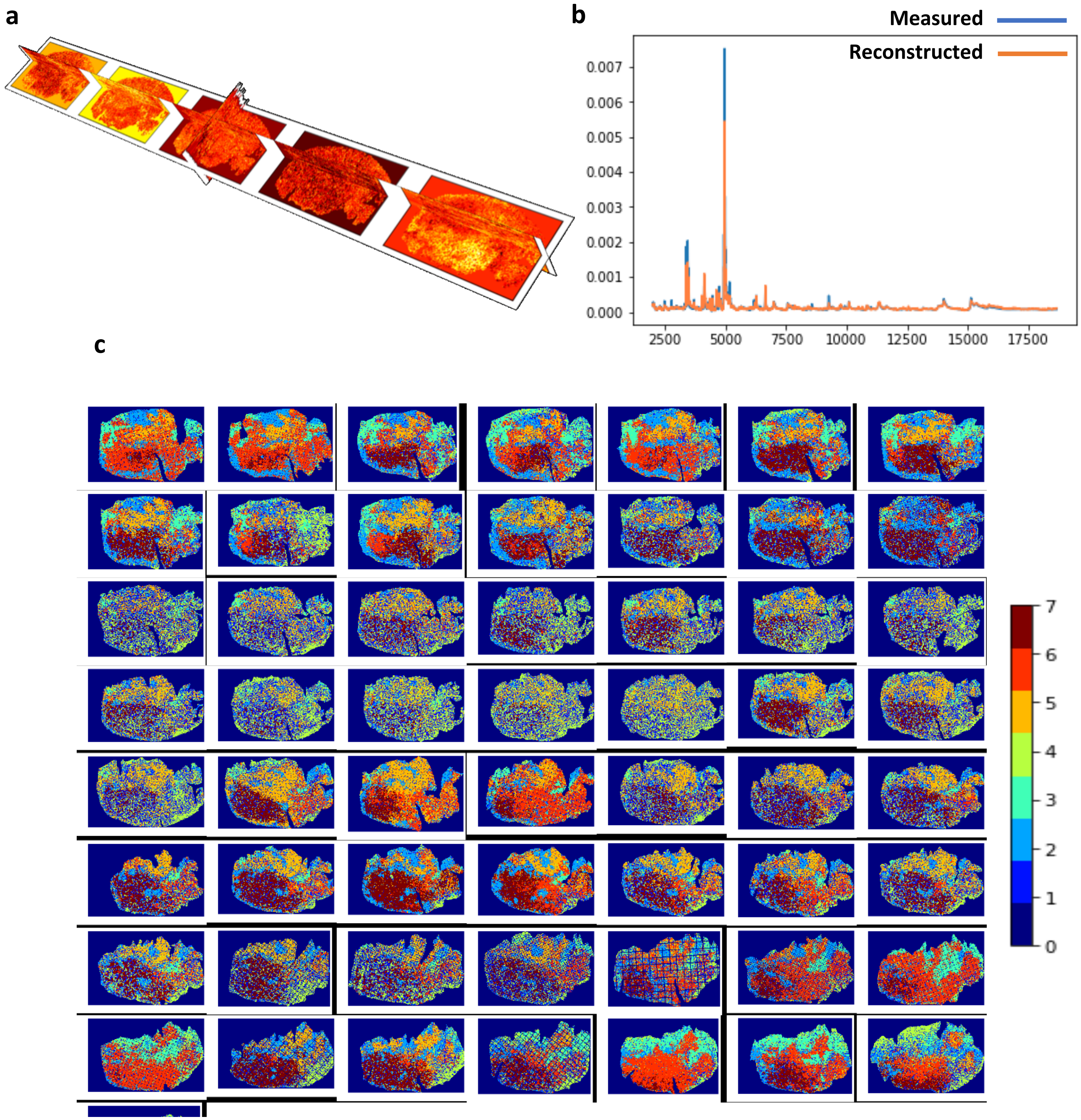

Supplementary Figure 9. 3D MALDI MSI dataset from 57 human OSCC tissue samples that were withheld for test analysis using the trained model shown in Figure S7: **a**. 3D distribution of the encoded features, **b**. overlay of the overall mean spectrum of both TIC normalized original (blue) and reconstructed (orange) full test dataset with mean squared error of 3.03×10^{-3}. **c**. clustering of encoded features using GMM (k=8) reveals molecular patterns (for example cluster#2 represents molecular phenotypes of defensins that are produced by the Neutrophils).

a

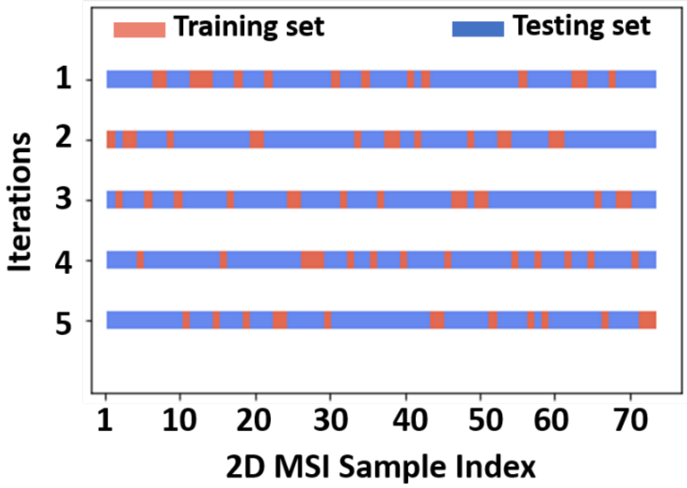

b

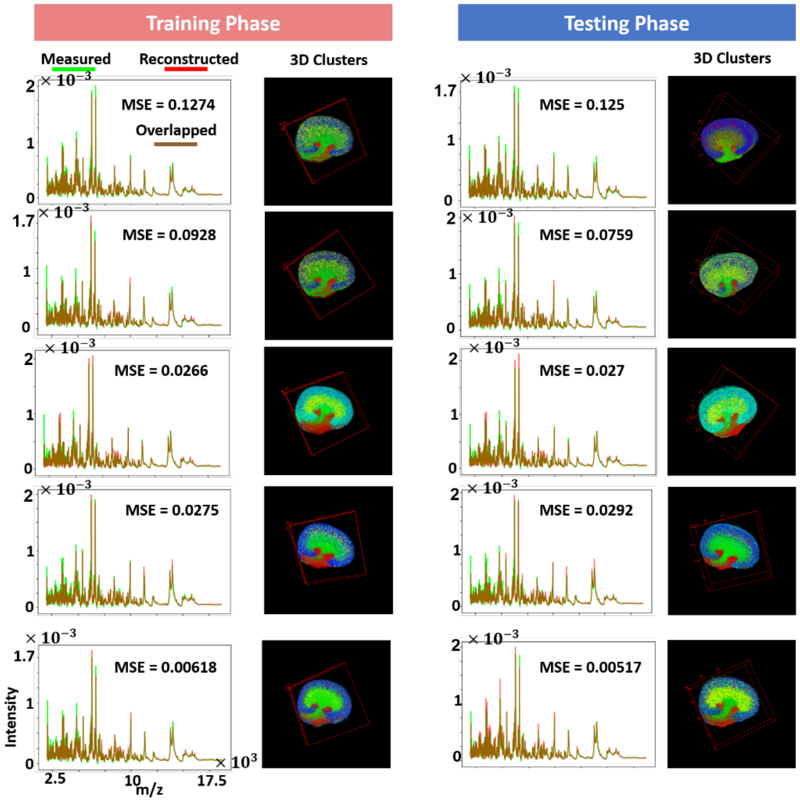

Supplementary Figure 10. Cross-validation analysis for the 3D MALDI MSI dataset of the mouse kidney (73 consecutive tissue sections): a. the full MSI dataset was randomly shuffled and split into a 20% training set and an 80% testing set, and this process was repeated 5 times. (b) For each iteration in the cross-validation (each row), the msiPL model was applied on the training set to optimize the artificial neural network and the trained model was then applied on the unseen test set. The original MSI data was completely reconstructed with a small mean squared error (MSE), and the overlay of the average spectrum of both TIC-normalized original and reconstructed data reveals close distribution. The learned non-linear manifold (encoded features) was clustered using GMM(k=8) which revealed distinct molecular patterns that reconcile with the kidney's anatomy. The trained model was robust and did not overfit the test set as it showed comparable performance to the training phase.

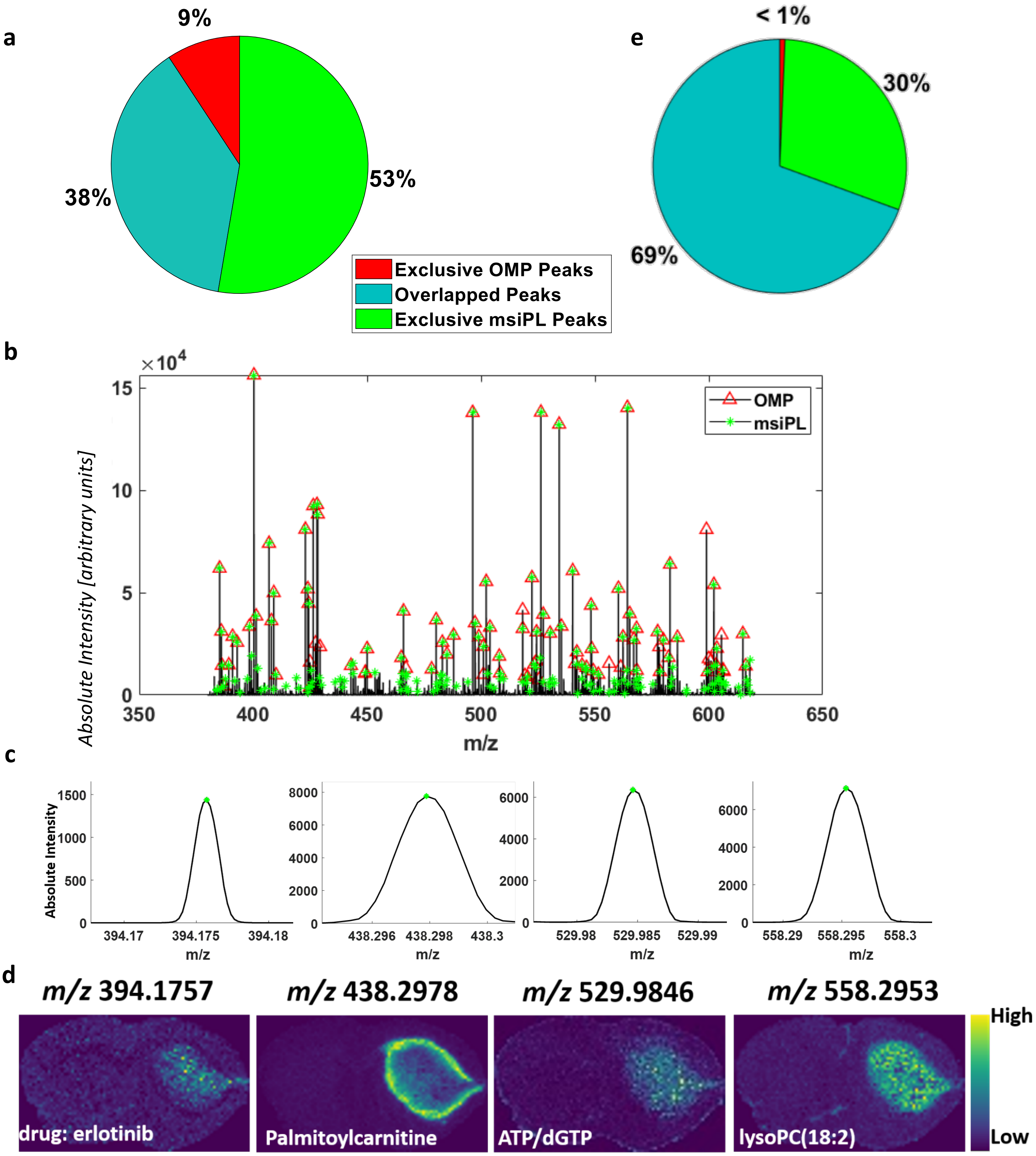

Supplementary Figure 11. Comparison of peak picking analysis on the 3D MALDI FT-ICR MSI PDX GBM dataset: (a-b) using msiPL on full spectral data and the orthogonal matching pursuit (OMP) algorithm on the mean spectrum (implemented in the commercial software of SCiLS Lab version 2020a (Bruker, Bremen, Germany)). (c-d) visualization of some of the peaks with biological relevance identified only by the manifold learning approach of msiPL but not by a classical approach of applying the OMP algorithm on the mean spectrum. (e) msiPL covered 99% of peaks identified by a more powerful approach of applying the OMP method on a wider range of spectra (thousands) but at cost of slow processing (Supplementary Table 7).

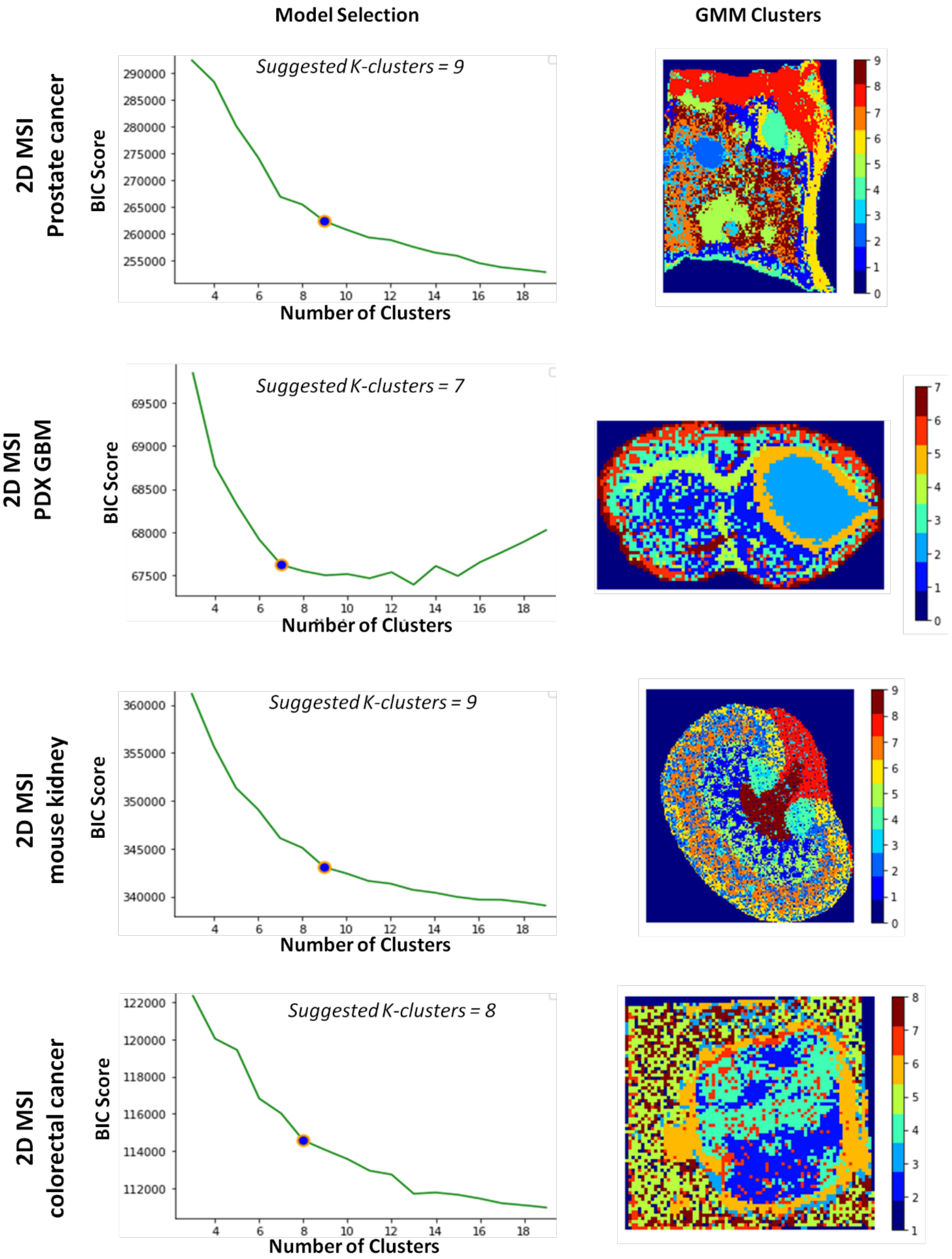

Supplementary Figure 12. Optimization-based selection of the number of K-clusters: Model selection of K-clusters using Bayesian information criterion (BIC) and the Kneedle algorithm to identify the optimum model at the point of maximum curvature (first column), and the spatial distribution of GMM clusters based on the suggested number clusters (second column).

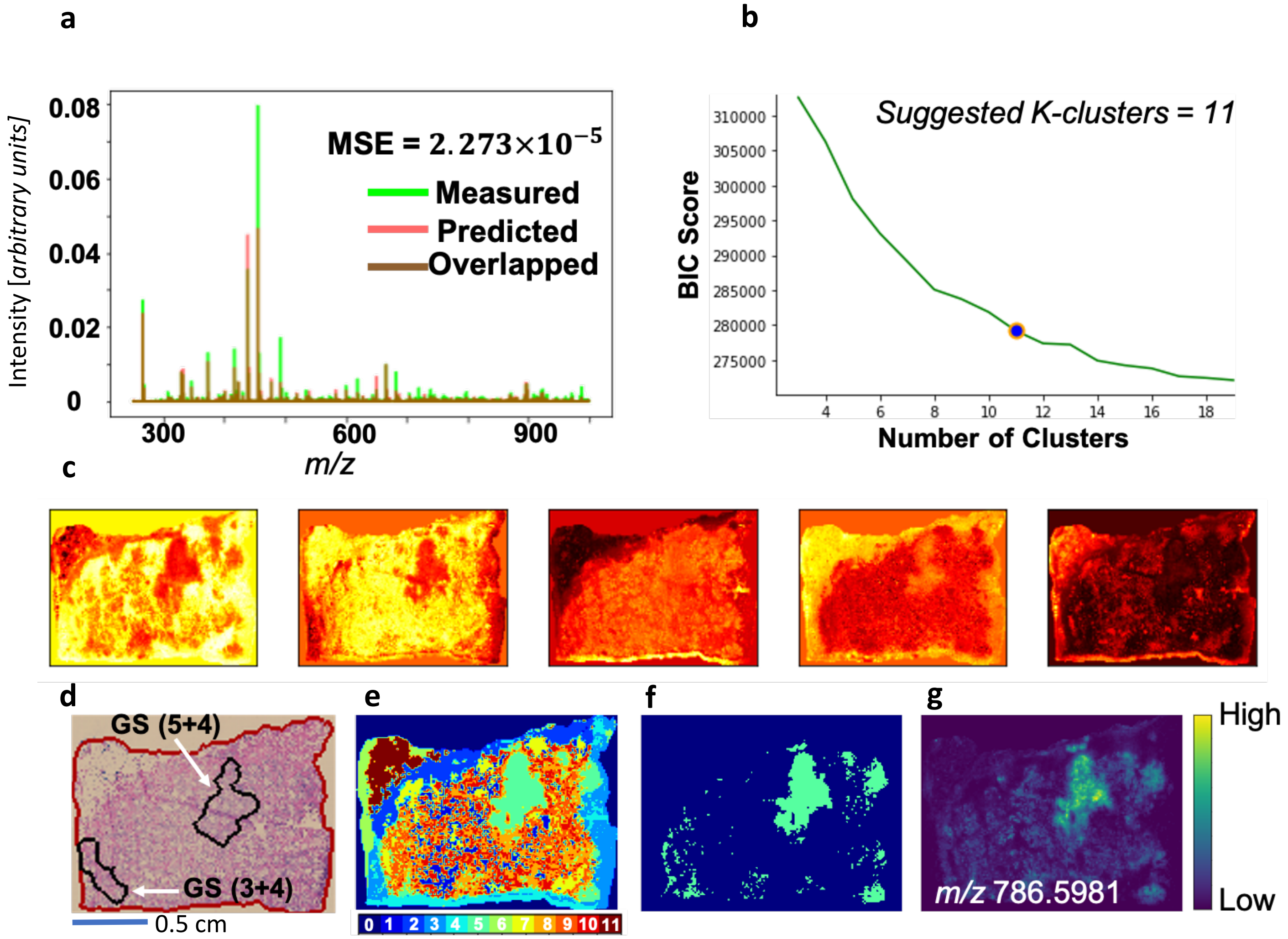

Supplementary Figure 13. Analysis of a test MALDI FT-ICR MSI dataset from prostate cancer tissue: **a.** Overlay of the mean spectrum of both TIC normalized original (green) and predicted (red) data with an overall mean squared error of 2.273×10^{-5}. **b.** Model selection of K-clusters to automatically cluster the encoded features shown in (c) using Bayesian information criterion (BIC) and the Kneedle algorithm. **d.** Histopathological annotation of the cancerous regions and associated Gleason score (GS). **e.** GMM-based clustering (K=11) of the encoded features (c) reveals a tumor cluster (f) that was found associated with a higher tumor grade region of GS (5+4). **g.** Spatial distribution of the highest correlated ion feature with the tumor cluster (f) was found at m/z 786.5981± 0.001 with a Pearson correlation coefficient of 0.746.

Supplementary Table 1. FT-ICR MSI prostate dataset: highly correlated *m/z* ion peaks with the prostate molecular-based tumor cluster

m/z experimental	correlation	*m/z* Identity	Molecular formula	Adduct	*m/z* calculated	Database	Error (ppm)
739.4664	0.7015	PA	$C_{39}H_{73}O_8P$	[M+K]	739.4675	HMDB	1.44
763.4662	0.6852	PA	$C_{41}H_{73}O_8P$	[M+K]	763.4675	HMDB	1.66
737.4506	0.6669	PA	$C_{39}H_{71}O_8P$	[M+K]	737.4518	HMDB	1.65
985.5567	0.6519	PIP(P-42:6)	$C_{51}H_{88}O_{15}P_2$	[M+H-H2O]+	985.5566	LipidMaps	-0.14
761.5867	0.6472						
760.5827	0.6462						
738.4548	0.6462	PI-Cer(t30:2)	$C_{36}H_{68}NO_{12}P$	[M+H]+	738.4552	LipidMaps	0.53
786.5981	0.6441	PC(O-34:0(OH))	$C_{42}H_{86}NO_8P$	[M+Na]+	786.5983	LipidMaps	0.29
		PE(O-37:0(OH))	$C_{42}H_{86}NO_8P$	[M+Na]+	786.5983	LipidMaps	0.29

Supplementary Table 2. Highly correlated *m/z* ion peaks with the tumor rim (Cluster#4) of the FT-ICR MSI PDX mouse brain dataset

m/z experimental	correlation	*m/z* Identity	Molecular formula	Adduct	*m/z* calculated	Database	Error (ppm)
438.2979	0.515	Palmitoylcarnitine	$C_{23}H_{45}NO_4$	[M+K]+	438.2980	HMDB	0.27
464.3132	0.484	Carnitine	$C_{25}H_{47}NO_4$	[M+K]+	464.3137	HMDB	1.01
400.3419	0.464	Palmitoylcarnitine	$C_{23}H_{45}NO_4$	[M+H]+	400.3421	HMDB	0.59
428.3734	0.463	Stearoylcarnitine	$C_{25}H_{49}NO_4$	[M+H]+	428.3734	HMDB	0.08
401.3454	0.459						
398.3259	0.458	9-Hexadecenoylcarnitine	$C_{23}H_{43}NO_4$	[M+H]+	398.3265	HMDB	1.47

Supplementary Table 3. Highly correlated *m/z* ion peaks with the tumor region1 (Cluster#2) of the FT-ICR MSI PDX mouse brain dataset

m/z experimental	correlation	*m/z* Identity	Molecular formula	Adduct	*m/z* calculated	Database	Error (ppm)
567.9409	0.733						
605.8966	0.73						
589.923	0.728						
529.9846	0.718	ATP/dGTP	$C_{10}H_{16}N_5O_{13}P_3$	[M+Na]+	529.9850	HMDB	0.69
589.9102	0.711						
545.959	0.707	ATP/dGTP	$C_{10}H_{16}N_5O_{13}P_3$	[M+K]+	545.9589	HMDB	-0.18
464.9473	0.704						
567.9285	0.701						
583.9147	0.7						
605.8842	0.699						

Supplementary Table 4. Highly correlated *m/z* ion peaks with the tumor region2 (Cluster#8) of the FT-ICR MSI PDX mouse brain dataset

m/z experimental	correlation	*m/z* Identity	Molecular formula	Adduct	*m/z* calculated	Database	Error (ppm)
558.2953	0.743	LysoPC(18:2)	C26H50NO7P	[M+K]+	558.2956	HMDB	0.62
520.3397	0.737	LysoPC(18:2)	C26H50NO7P	[M+H]+	520.339766	HMDB	0.13
468.3083	0.729	LysoPC(14:0) / LysoPE	C22H46NO7P	[M+H]+	468.3084	HMDB	0.35
414.3579	0.727	Heptadecanoyl carnitine	C24H47NO4	[M+H]+	414.3577	HMDB	-0.28
521.3428	0.717						
425.3453	0.716						
424.3421	0.713	Linoelaidyl carnitine or Linoleyl carnitine	C25H45NO4	[M+H]+	424.3421	HMDB	0.08
494.3239	0.709	Cervonyl carnitine	C29H45NO4	[M+Na]+	494.3240	HMDB	0.36
		LysoPC(16:1)	C24H48NO7P	[M+H]+	494.3241	HMDB	0.44
532.28	0.7	LysoPC(16:1)	C24H48NO7P	[M+K]+	532.2799	HMDB	0.00
426.3573	0.697	Carnitine	C25H47NO4	[M+H]+	426.3577	HMDB	1.14

Supplementary Table 5. Different parameter settings of the neural network model (tested on a 2D MALDI MSI data of PDX mouse brain model of glioblastoma)

Design	#Hidden Layers	#Computational Neurons per Layer	#Model Parameters	Running Time (Minutes)	MSE of Original and Reconstructed Data
Architecture1	3 Layers	50-5-50	2,146,421	3.1	6.52×10^{-4}
Architecture2	3 Layers	512-3-512	21,779,723	3.2	4.72×10^{-4}
Architecture3	3 Layers	512-5-512	21,782,807	3.6	4.56×10^{-4}
Architecture4	3 Layers	512-15-512	21,798,227	3.6	3.47×10^{-4}
Architecture5	3 Layers	5000-5-512	212,536,271	8.27	2.82×10^{-4}
Architecture6	5 Layers	3000-512-5-512-3000	120,568,023	5.8	2.02×10^{-4}
Architecture7	7 Layers	5000-1000-512-5-512-1000-5000	232,502,023	8.4	2.28×10^{-4}

Supplementary Table 6. Running time of training the msiPL on the GPU compared to the CPU.

Dataset	msiPL on CPU	msiPL on GPU
2D FT ICR MSI of human prostate cancer (12,716 spectra; 730,403 m/z bins)	149.8 minutes	40 minutes
2D MSI of mouse kidney (18,536 spectra; 7671 m/z variables)	25.2 minutes	8.6 minutes

Supplementary Table 7. Running Time for peak picking using commercial software compared to msiPL

Dataset	Running time for only peak picking step using OMP algorithm implemented in SCiLS 2019c (Bruker, Germany)	The entire msiPL analysis (not just the peak learning step)
2D FT ICR MSI of human prostate cancer (12,716 spectra; 730,403 m/z bins) *<br>*sampling every 20th spectrum for OMP analysis.	4 hours and 50 minutes	40 minutes
3D MALDI MSI of a PDX mouse brain model of glioblastoma (14,833 spectra; 661,402 m/z bins)+<br>+sampling every 5th spectrum for SCiLS OMP.	10 hours and 39 minutes	Training phase: 3.6 minutes Testing phase: 8 seconds.<br><br>Note: 3D MSI dataset is analyzed using msiPL using training/testing strategy as explained in the manuscript.

Supplementary Table 8. Comparison of mean squared error (MSE) of MSI data reconstruction using different methods

	PCA	Memory Efficient PCA	DWT + PCA	msiPL
FT ICR MSI Prostate Dataset	1.886×10^{-2}	2.583×10^{-2}	1.81×10^{-2}	2.42×10^{-5}
FT ICR MSI GBM Dataset	8.405×10^{-3}	7.018×10^{-3}	8.6×10^{-3}	4.5×10^{-4}

Supplementary References

1. Randall, E. C. *et al.* Molecular characterization of prostate cancer with associated Gleason score using mass spectrometry imaging. *Mol. Cancer Res.* (2019). doi:10.1158/1541-7786.MCR-18-1057

2. Randall, E. C. *et al.* Integrated mapping of pharmacokinetics and pharmacodynamics in a patient-derived xenograft model of glioblastoma. *Nat. Commun.* **9**, (2018).

3. Oetjen, J. *et al.* Benchmark datasets for 3D MALDI- and DESI-imaging mass spectrometry. *Gigascience* **4**, 20 (2015).
