# Supplementary material for: Peak learning of mass spectrometry imaging data using artificial neural networks
Source: Nat Commun. 2021 Sep 20;12:5544. doi: 10.1038/s41467-021-25744-8 (PMC8452737; doi:10.1038/s41467-021-25744-8)
Supplement: Supplementary file 3 — Description of Additional Supplementary Files [file 41467_2021_25744_MOESM3_ESM.pdf]

Description of Additional Supplementary Files

File Name: Supplementary Data 1
Description: Reduced list of *m/z* values revealed as main determinants of the molecular patterns captured in the latent space in the analysis of 5 different datasets.

File Name: Supplementary Data 2
Description: Peak list from the different cross-validation models.

File Name: Supplementary Data 3
Description: List of peaks identified by both msiPL and the OMP of the 3D MALDI MSI PDX GBM dataset.
