# Supplementary material for: Peak learning of mass spectrometry imaging data using artificial neural networks
Source: Nat Commun. 2021 Sep 20;12:5544. doi: 10.1038/s41467-021-25744-8 (PMC8452737; doi:10.1038/s41467-021-25744-8)
Supplement: Supplementary file 7 — Reporting Summary [file 41467_2021_25744_MOESM7_ESM.pdf]

nature research

Corresponding author(s): Nathalie Y.R. Agar

Last updated by author(s): Jul 19, 2021

Reporting Summary

Nature Research wishes to improve the reproducibility of the work that we publish. This form provides structure for consistency and transparency in reporting. For further information on Nature Research policies, see our Editorial Policies and the Editorial Policy Checklist.

Statistics

For all statistical analyses, confirm that the following items are present in the figure legend, table legend, main text, or Methods section.

n/a	Confirmed	
☐	☒	The exact sample size (*n*) for each experimental group/condition, given as a discrete number and unit of measurement
☐	☒	A statement on whether measurements were taken from distinct samples or whether the same sample was measured repeatedly
☒	☐	The statistical test(s) used AND whether they are one- or two-sided<br>*Only common tests should be described solely by name; describe more complex techniques in the Methods section.*
☒	☐	A description of all covariates tested
☒	☐	A description of any assumptions or corrections, such as tests of normality and adjustment for multiple comparisons
☒	☐	A full description of the statistical parameters including central tendency (e.g. means) or other basic estimates (e.g. regression coefficient) AND variation (e.g. standard deviation) or associated estimates of uncertainty (e.g. confidence intervals)
☒	☐	For null hypothesis testing, the test statistic (e.g. *F*, *t*, *r*) with confidence intervals, effect sizes, degrees of freedom and *P* value noted<br>*Give P values as exact values whenever suitable.*
☐	☒	For Bayesian analysis, information on the choice of priors and Markov chain Monte Carlo settings
☐	☒	For hierarchical and complex designs, identification of the appropriate level for tests and full reporting of outcomes
☐	☒	Estimates of effect sizes (e.g. Cohen's *d*, Pearson's *r*), indicating how they were calculated

Our web collection on statistics for biologists contains articles on many of the points above.

Software and code

Policy information about availability of computer code

Data collection	Two datasets were acquired in our laboratories by MALDI FT ICR MSI using 9.4 Tesla SolariX XR (Bruker Daltonics, Billerica, MA) and exported in imzML file format using SCiLS Lab(2019c, Bruker Daltonics, Germany). The three other MSI datasets were acquired by MALDI TOF and DESI Orbitrap and publicly available for download as explained in [Oetjen et al, GigaScience,4(1), 2015].
Data analysis	The machine learning model was implemented using open source platforms of Python(3.6.4) with keras(2.1.5-tf) and tensorflow(version 1.8.0), numpy(1.14.2), sklearn(0.19.1), scipy(1.0.0), matplotlib(3.0.2), Kneed (0.6.0), and h5py(2.7.1). Data analysis was performed on our PC workstation (Intel Xenon 3.3GHz, 512 GB RAM, 64-bit Windows, 2 GPUs NVIDIA TITAN Xp). Peak picking was performed using SCiLS Lab (2020a, Bruker Daltonics, Germany). The source code is available on GitHyb via this link: https://github.com/wabdelmoula/msiPL.git

For manuscripts utilizing custom algorithms or software that are central to the research but not yet described in published literature, software must be made available to editors and reviewers. We strongly encourage code deposition in a community repository (e.g. GitHub). See the Nature Research guidelines for submitting code & software for further information.

Data

Policy information about availability of data

All manuscripts must include a data availability statement. This statement should provide the following information, where applicable:

- Accession codes, unique identifiers, or web links for publicly available datasets
- A list of figures that have associated raw data
- A description of any restrictions on data availability

Results of Figures 5-7 and Supplementary Figures S2-S10 are associated with public 3D MSI datasets from [Oetjen et al, GigaScience,4(1), 2015]. Results of Figures 2-4 and Supplementary Figures S1, S11 and S13 are associated with two MSI datasets that were acquired in our laboratories (human prostate and PDX mouse brain

model of glioblastoma), and are available on the NIH Common Fund's National Metabolomics Data Repository (NMDR) Metabolomics Workbench (https://www.metabolomicsworkbench.org) under project id (PR001171) with doi http://dx.doi.org/10.21228/M8BM4Q

Field-specific reporting

Please select the one below that is the best fit for your research. If you are not sure, read the appropriate sections before making your selection.

☒ Life sciences ☐ Behavioural & social sciences ☐ Ecological, evolutionary & environmental sciences

For a reference copy of the document with all sections, see nature.com/documents/nr-reporting-summary-flat.pdf

Life sciences study design

All studies must disclose on these points even when the disclosure is negative.

Sample size	The computational model was trained on thousands of high dimensional datapoints (i.e. spectra) sampled from an entire 2D tissue surface, and the sample size of the MSI data size is as following: 2D prostate data (12,716 spectra and 730,403 m/z bins), 3D PDX data (training: 3,570 spectra; testing: 11,263 spectra; and 661,402 m/z bins), 3D mouse kidney data(training: 18,536 spectra;, testing: 1,342,294 spectra; ; 7671 m/z bins), 3D Colorectal adenocarcinoma data(training: 5,694 spectra; testing: 142,350; spectra; 8,073 m/z bins), and 3D oral squamous cell carcinoma (training: 12,875 spectra; testing: 815,683 spectra; 7,6665 m/z bins). These sample sizes were sufficient to show learning stability and model convergence that resulted in comparable performances in manifold learning and minimizing the reconstruction error in both training and testing data (e.g. see distribution of model convergence in Figures 2.a and 3.a as well as in supplementary figures Figure S2.a, S4.a, S7.a).
Data exclusions	No data were excluded.
Replication	The robustness of the computational model was tested on MSI data acquired at different centers from different biological systems and acquired using different mass spectrometers equipped with different ionization methods and different mass analyzers. The model stability was also tested using cross-validation analysis as explained in the main text.
Randomization	The model was optimized on a randomly shuffled spectral batches of size 128 spectra/batch. In case of 3D MSI data, the model was trained on a set of spectra acquired from a full 2D tissue section (first section in the 3D stack was arbitrary chosen) and the test was done on the withheld 3D MSI data.
Blinding	Not relevant to the machine learning. However, our neural network model is well regularized (batch normalization and kullback-Leibler divergence) to stabilize the learning performance and avoid overfitting and this was assessed on the performance of a new unseen data.

Reporting for specific materials, systems and methods

We require information from authors about some types of materials, experimental systems and methods used in many studies. Here, indicate whether each material, system or method listed is relevant to your study. If you are not sure if a list item applies to your research, read the appropriate section before selecting a response.

Materials & experimental systems

n/a	Involved in the study
☒	☐ Antibodies
☒	☐ Eukaryotic cell lines
☒	☐ Palaeontology and archaeology
☒	☐ Animals and other organisms
☒	☐ Human research participants
☒	☐ Clinical data
☒	☐ Dual use research of concern

Methods

n/a	Involved in the study
☒	☐ ChIP-seq
☒	☐ Flow cytometry
☒	☐ MRI-based neuroimaging
